# Psychopathological and temperamental features of Late Onset versus Early Onset Bipolar Disorder

**DOI:** 10.1192/j.eurpsy.2022.422

**Published:** 2022-09-01

**Authors:** L. Orsolini, L. Ferretti, M. Fiorani, D. Rocchetti, V. Salvi, U. Volpe

**Affiliations:** 1 Polytechnic University of Marche, Department Of Clinical Neurosciences/dimsc, School Of Medicine, Unit Of Psychiatry, Ancona, Italy; 2 Unit of Clinical Psychiatric, Polytechnic University of Marche, Ancona, Italy, Department Of Neurosciences/dimsc, Ancona, Italy

**Keywords:** LOBD, EOBD, bipolar disorder, temperament

## Abstract

**Introduction:**

Age at onset of type-I bipolar disorder (BD-I) typically averages 12-24 years, is older among patients with type-II-BD (BD-II), even though generally before 50-years-old (EOBD). Clinical observation of late-onset BD (LOBD) posed some questions regarding a differential phenotypic/psychopathological manifestations and affective temperaments between LOBD vs EOBD.

**Objectives:**

A case-control pilot-study was carried out to investigate psychopathological, clinical and temperamental features of a psychogeriatric cohort of LOBD and EOBD subjects.

**Methods:**

Out of 74 enrolled patients, 64 patients (31 EOBD, 33 LOBD) were included and administered an ad hoc socio-demographic datasheet, BPRS, CGI, GAF, HAM-D, GDS, MSRS, MRS, MOCA and TEMPS-M.

**Results:**

LOBD is significantly associated with higher rates of BD-II diagnosis (X2 = 26.1, p<.001), depressive (p=0.05) and mixed states (p=0.011), higher comorbid anxiety levels and depressive affective temperament (p<.001); while clinical manifestations of geriatric EOBD is significantly associated with higher endocrinological (X2 = 7.815, p=.005) and metabolic comorbidity (X2 = 6.896, p=.009), a diagnosis of BD-I, manic episodes and hyperthymic (p=.001) affective temperaments. GDS and MSRS total scores were significantly higher in LOBD (respectively, p<.001 and p=.008).

**Conclusions:**

Further studies with larger sample sizes and a control group should verify whether LOBD is a distinct psychopathological entity from EOBD and evaluate differences (if any) in terms of prognosis and treatment between EOBD and LOBD.

**Disclosure:**

No significant relationships.

